# An Integrated Transcriptome Analysis Reveals IGFBP7 Upregulation in Vasculature in Traumatic Brain Injury

**DOI:** 10.3389/fgene.2020.599834

**Published:** 2021-01-11

**Authors:** Jianhao Wang, Xiangyi Deng, Yuan Xie, Jiefu Tang, Ziwei Zhou, Fan Yang, Qiyuan He, Qingze Cao, Lei Zhang, Liqun He

**Affiliations:** ^1^Key Laboratory of Post-Neuroinjury Neuro-Repair and Regeneration in Central Nervous System, Department of Neurosurgery, Tianjin Medical University General Hospital, Tianjin Neurological Institute, Ministry of Education and Tianjin City, Tianjin, China; ^2^Key Laboratory of Ministry of Education for Medicinal Plant Resource and Natural Pharmaceutical Chemistry, National Engineering Laboratory for Resource Developing of Endangered Chinese Crude Drugs in Northwest of China, College of Life Sciences, Shaanxi Normal University, Xi’an, China; ^3^Trauma Center, First Affiliated Hospital of Hunan University of Medicine, Huaihua, China; ^4^Precision Medicine Center, The Second People’s Hospital of Huaihua, Huaihua, China; ^5^Department of Immunology, Genetics and Pathology, Uppsala University, Uppsala, Sweden

**Keywords:** traumatic brain injury, vasculature, endothelial cell, IGFBP7, TGFβ

## Abstract

Vasculature plays critical roles in the pathogenesis and neurological repair of traumatic brain injury (TBI). However, how vascular endothelial cells respond to TBI at the molecular level has not been systematically reviewed. Here, by integrating three transcriptome datasets including whole cortex of mouse brain, FACS-sorted mouse brain endothelial cells, and single cell sequencing of mouse brain hippocampus, we revealed the key molecular alteration of endothelial cells characterized by increased Myc targets and Epithelial-Mesenchymal Transition signatures. In addition, immunofluorescence staining of patients’ samples confirmed that IGFBP7 was up-regulated in vasculature in response to TBI. TGFβ1, mainly derived from microglia and endothelial cells, sufficiently induces IGFBP7 expression in cultured endothelial cells, and is significantly upregulated in response to TBI. Our results identified IGFBP7 as a potential biomarker of vasculature in response to TBI, and indicate that TGFβ signaling may contribute to the upregulation of IGFBP7 in the vasculature.

## Introduction

Traumatic brain injury (TBI), one of the leading cause of morbidity and disability, accounts for 30% of all injury-related deaths (Maas et al., 2008). It has been estimated that in the United States, an estimated 1.7 million people experience TBI annually, and 5.3 million people suffer TBI-related complications and sequela including long-term neurological and psychiatric disorders, chronic inflammation, and chronic traumatic encephalopathy (Nguyen et al., 2016; Jassam et al., 2017). TBI is a complicated pathophysiological process that can be divided into primary and secondary brain injuries (Sun et al., 2017). Primary injury is the direct damage of neural tissue caused by mechanical effect occurring at the moment of trauma (Silverberg et al., 2019). By contrast, secondary injury is the indirect injurious biochemical cascade initiated by primary insult, significantly contributing to the aggravation and high mortality of TBI (Bramlett and Dietrich, 2015; Silverberg et al., 2019). Secondary brain injury is mediated by several cellular and molecular pathways including excitotoxicity, inflammation, oxidative stress, and energy failure, and cerebral vasculature is a critical player to regulate these pathological processes (Jassam et al., 2017; Salehi et al., 2017; Simon et al., 2017). There is an increasing interest in the role that brain vasculature plays in the pathogenesis of TBI. Direct disruption of cerebral vasculature at the time of head impact leads to hemorrhage and blood flow abnormalities immediately after trauma, and dysfunction of vasculature leads to additional insults such as hypoxemia, hypoxia, hypo-perfusion, ischemia, and blood brain barrier (BBB) breakdown (Jullienne et al., 2016).

BBB is a specialized vascular structure in brain that maintains homeostasis and regulates the movement of molecular and cells across the brain vasculature (Yeoh et al., 2013). BBB disruption, as assessed by cerebrospinal fluid/serum albumin quotient, is found in 44% of non-penetrating TBI patients (Ho et al., 2014). Alteration of the vasculature in TBI causes vasogenic edema at both lesion and surrounding tissues resulting in tissue swelling and elevated intracranial pressure, contributing to about 50% of death in severe head injury (Marmarou, 2003). Moreover, increased vascular permeability observed in TBI patient allows harmful molecular and blood toxins into brain, which may lead to neuronal damage and long-lasting functional deficits (Badaut et al., 2011). In addition, up-regulation of cytokines/chemokines and leukocyte adhesion molecules in vascular endothelial cells (EC) augment inflammation and further increase the risk of edema formation and neuronal dysfunction (Ziebell and Morganti-Kossmann, 2010; Simon et al., 2017). Therefore, vasculature plays a critical role in the pathogenesis of TBI, and therapeutical manipulation of vasculature may represent a potential way for TBI treatment. However, how vascular EC respond to TBI at the molecular level has not been systematically reviewed.

Here, we integrated three transcriptome datasets in which the responses of whole cortex, FACS-sorted ECs, and isolated single ECs to brain injury were documented. Unbiased comparison by aligning three data sets to the same mouse genome revealed the key molecular alteration of ECs in response to brain injury. In addition, IGFBP7 was identified as a potential biomarker of vasculature in TBI pathogenesis.

## Materials and Methods

### Sequencing Data Collection

The original sequencing data from three independent studies were obtained from the NCBI Sequence Read Archive (SRA) database^1^, including the mouse whole brain cortex RNAseq data (SRP072117, six samples), the FACS-sorted mouse brain endothelial RNAseq data (SRP100777, six samples), and the mouse brain hippocampus single cell RNAseq data (SRP113600, six samples). Each sample contains about 40–60 million RNA sequence reads.

### Bulk RNAseq Data Analysis

The RNAseq sequencing data from the brain cortex and FACS-sorted EC were aligned to the mouse genome assembly (GRCm38) obtained from the Ensembl database using the TopHat2 software (version 2.1.1, with the following parameters: tophat2 -p 8 –keep-fasta-order –GTF < reference.gtf >< reference.genome >< read1.fastq >< read2.fastq >) (Kim et al., 2013). To quantify the differential expression between TBI and control groups, the default cuffdiff tests were performed using the Cufflinks tool (version 2.2.1, with the following parameters: cuffdiff -p 4 < reference.gtf >< control1.bam,control2.bam,control3.bam >< TBI1.bam,TBI2.bam,TBI3.bam >) (Trapnell et al., 2012). The genes with statistical multiple-test corrected (Benjamini-Hochberg) *p*-value smaller than 0.05 and more than 2-fold difference were selected as significant differentially expressed genes.

To compare the gene expression levels among different samples, heatmap visualization was performed using the pheatmap packages (version 1.0.12) with ward.D2 method. The genes were clustered using the Pearson correlation distance and the samples were clustered using the Euclidean distance.

### Single Cell RNAseq Data Analysis

The single cell RNAseq data were processed using the Drop-seq tools (version 2.3.0)^2^ with default parameters as described in the paper (Arneson et al., 2018). The fastq files were aligned to the mouse genome assembly (GRCm38) and the digital expression counts for each cell were quantified using the default parameters. The single cell expression counts data were then imported into the Seurat package (version: 3.1.1) for cell type clustering and cluster marker identifications (Butler et al., 2018).

### Gene Set Variation Analysis (GSVA)

The GSVA was performed using the R GSVA package (version 1.32.0). To identify the gene sets with significant changes, the 50 hallmark gene sets from the MSigDB collections were tested^3^ using the R limma package (version 3.40.6). The gene sets with statistical multiple-test (Benjamini-Hochberg) corrected *p*-value smaller than 0.05 were identified as significant.

### Human TBI Sample Collection

The brain tissue was collected from the Department of Neurosurgery, Tianjin Medical University General Hospital, Tianjin 300052, PR China. The patient was an adult male. The preoperative Glasgow Coma Scale (GCS) was 11 points without underlying disease. The written informed consent from the patient was made before the surgery.

During the operation, the contusion tissue was extracted from the contusion area by bipolar electrocoagulation, and the brain contusion tissue was marked as TBI group. The control specimens were taken from the non-functional area about 1.5 cm from the tumor boundary of the glioblastoma patient. After removing the specimens, they were stored at -80°C. All human sample experiments were approved by the local hospital ethics committee.

### Immunofluorescence Staining

After the specimens were taken out from -80°C, they were quickly embedded in optimal cutting temperature (O.C.T., Sakura, Oakland, CA, United States). Then, the specimens were cut into a thick coronal section of 6 μm, and immunofluorescence stained to determine the IGFBP7 and CD31 expression in the coronal brain section. The slices were first fixed in ice-cold methanol for 10 min and washed with phosphate buffered saline (PBS) for 10 min. Blocked with 3% BSA (Invitrogen) for 1 h at room temperature (RT). Then, the sections were incubated with the primary antibody against IGFBP7 (1:200, ab74169, Abcam) and CD31 (1:500, MA3105, Invitrogen) overnight at 4°C. The slides were washed three times with PBS plus 0.2% Tween (Sigma) and incubated with the secondary antibody Goat anti-Rabbit Alexa 555 (1:400, cat A-21428, Invitrogen) and Goat anti-Armenian hamster Alexa 488 (1:400, ab173003, Abcam) for 1 h at RT. After washing with PBS plus 0.2% Tween three times, the sections were mounted in Fluoromount (F4680, Sigma) containing 0.1% hoechst 33258 (14530, Sigma). Images were acquired by fluorescence microscope (Zeiss, Germany).

### *In vitro* Stimulation of EC

Murine brain EC (bEND.3) were cultured in Dulbecco’s modified Eagle’s medium (DMEM) (Gibco) supplemented with 10% fetal bovine serum (Biological Industries) and 1% 100 mM Sodium Pyruvate (Gibco). Human umbilical vein EC (HUVECs) were maintained up to passage 14 in the same culture medium as described above. The bEND.3 cells and HUVECs were starved in DMEM with 1% FBS or 0.5% FBS, respectively, overnight and stimulated with TGFβ1 (2 ng/mL; Peprotech) for 72 h, VEGFA (50 ng/mL; Peprotech) for 72 h, or cobalt chloride (400 μM CoCl_2_; Sigma Aldrich) for 24 h, respectively. The experiment was performed duplicates and repeated three times for both bEND.3 cells and HUVECs.

### Quantitative Real-Time Q-PCR

Total cell RNA was extracted with the RNeasy kit (Qiagen) and reverse-transcribed with the PrimeScript RT Master Mix (Takara). qPCR was performed on a Thermal Cycler iQ5 Multicolor Real-Time PCR Detection system (Bio-Rad) using TB Green Premix Ex taqTM II (Takara) and intron-spanning, gene-specific primers as listed below: mouse *Hprt* (forward: CAGTCCCAGCGTCGTGATTA, reverse: TGGCCTCCCATCTCCTTCAT); mouse *Igfbp7* (forward: CTG GTGCCAAGGTGTTCTTGA, reverse: CTCCAGAGTGATCC CTTTTTACC); human *HPRT* (forward: CTTTGCTGACCT GCTGGATT, reverse: TCCCGTGTTGACTGGTCATT); human *IGFBP7* (forward: GCGAGCAAGGTCCTTCCATA, reverse: TCTGAATGGCCAGGTTGTCC). Gene expression was normalized to the house keeping gene *HPRT*(*Hprt*).

## Results

### Identification of Differentially Expressed Genes in TBI

In order to identify the genes that are differentially expressed in brain in TBI condition, we reanalyzed and integrated the data from three transcriptome sequencing profiling approaches (Table 1). These include one mouse whole cortex RNAseq study (Zhong et al., 2016), one mouse brain FACS-sorted endothelial RNAseq study (Munji et al., 2019), and one mouse brain single cell RNAseq study (Arneson et al., 2018). All three studies were performed with mouse TBI models and screened the gene expression changes at 24 h after TBI. The three datasets were processed differently in their corresponding studies. To achieve an unbiased comparison, we obtained the original sequence data for each sample and aligned them to the same mouse genome reference (Supplementary Table 1). Differentially expressed genes were identified by comparing the TBI and control in the respective studies. The complete gene analysis results from each of the three studies are listed in the supplements (whole cortex RNAseq study in Supplementary Table 2; FACS-sorted EC study in Supplementary Table 3; single cell RNAseq study in Supplementary Table 4).

**TABLE 1:** Summary of the characteristics of the three studies.

Study	Species and age	Sample	Comparing samples	TBI types; Severity; and time points	Parameters	Methods	Total reads
Zhong et al., 2016	C57BL/6 mouse; 12 weeks	Brain whole cortex	Three TBI samples vs. three controls	CCI; Moderate; 24 h	Diameter: 3 mm, velocity: 5.0 m/s, depth: 2.0 mm, dwelling time:100 ms	RNAseq	319 million
Munji et al., 2019	Rosa-tdTomato mouse; 21 days	FACS purified mouse brain endothelial cells	Three TBI samples vs. three controls	CCI; Focal; 24 h	Diameter: 3 mm, velocity: 4.5 m/s, depth: 1.73 mm, dwelling time:150 ms	RNAseq	344 million
Arneson et al., 2018	C57BL/6 mouse; 10 weeks	Single-cell suspension from brain hippocampus	Three TBI samples vs. three controls	FPI; Mild; 24 h	Diameter: 1.5 mm, velocity: 4.5 m/s, fluid percussion pulse: 1.5–1.7 atm	scRNAseq	384 million

**The table lists the three transcriptome sequencing analyses we reintegrated. The similarities and differences of type of TBI, severity, age, time points, and type of mouse are shown in detail. CCI, controlled cortical impact; FPI, fluid percussion injury.**

In the whole cortex RNAseq study, 1,096 genes were tested as significantly differentially expressed (Supplementary Table 5). In the FACS-sorted EC study, 214 genes were identified (Supplementary Table 6).

For the single cell RNAseq study, all the qualified cells (*N* = 6,351, with 2,549 cells from the TBI samples and 3,370 cells from the control samples) were clustered and a pure endothelial cluster (*n* = 371) was identified (Supplementary Figure 1). Within the endothelial clusters, the comparison between the 185 cells in TBI samples and the 186 cells in the control samples yields 14 significantly differentially expressed genes (Supplementary Table 7).

### Comparison and Integration of the Three Studies

**FIGURE 1:**
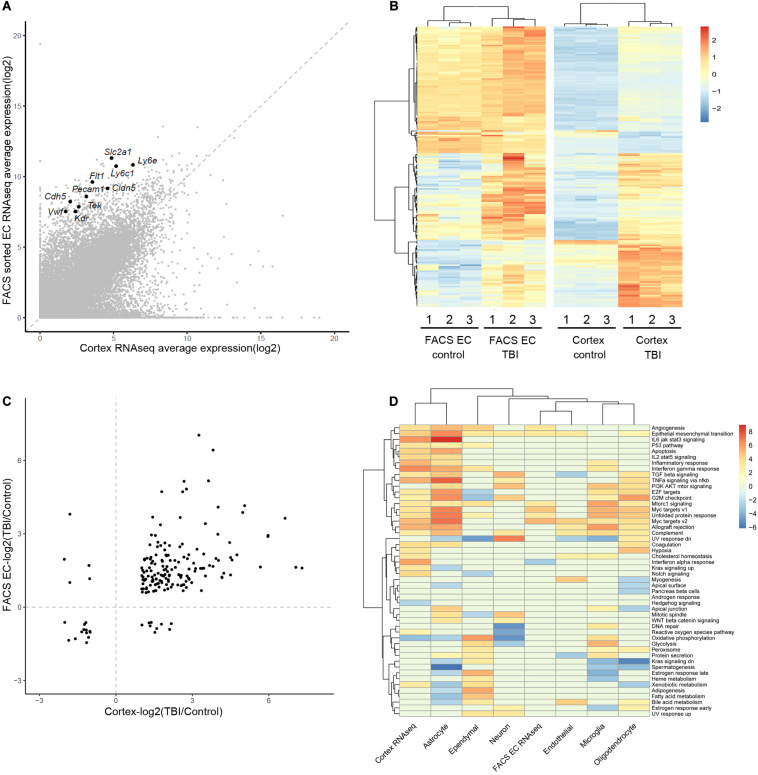
Comparison of the gene expression analysis results from the three studies. **(A)** Comparison of the average expression of whole cortex and FACS sorted EC RNAseq data. Ten well-known canonical EC markers are labeled. **(B)** Heatmap of the different expression of 184 DEGs in the two studies, using the pheatmap packages (version 1.0.12) with ward.D2 method. The color legend shows the relative expression. **(C)** Comparison of the log2 scaled fold change (TBI/Control) of the 184 DEGs in the two RNAseq studies. **(D)** Heatmap of the GSVA analysis of the three studies. The two RNAseq studies and the major cell types in the scRNAseq study are listed in the columns. The rows represent the 50 hallmark gene sets. The color legend shows the GSVA t statistics, and red color indicates up-regulation and blue color indicates down-regulation.

We focused on the brain EC to evaluate and compare the results from three studies. First, we compared the two RNAseq studies (whole cortex and FACS-sorted EC). For a list of 10 well-known canonical EC markers, they showed a wild range of expression in RNAseq data, however, they all display significant enrichment in the FACS sorted EC samples (around 20–100-folds’ enrichment) (Figure 1A). In the results of differentially expressed genes, there were 184 genes differentially expressed in both studies (Figure 1B and Supplementary Table 8). Among these 184 genes, 170 genes were regulated in the same direction (both were up-regulated or down-regulated), and only 14 (8%) showed different regulation directions (Figure 1C).

To identify the pathways that were affected during the TBI condition, Gene Set Variation Analysis (GSVA) was applied to each of the three datasets. The regulated hallmark gene sets in the two RNAseq studies as well as the major cell types in the single cell RNAseq studies were identified (Figure 1D). Myc targets and Epithelial-Mesenchymal Transition signature are enriched in samples from TBI in all three studies. Interestingly, both signatures are also enriched in tumor EC and associate with tumor angiogenesis (Lambrechts et al., 2018).

**FIGURE 2:**
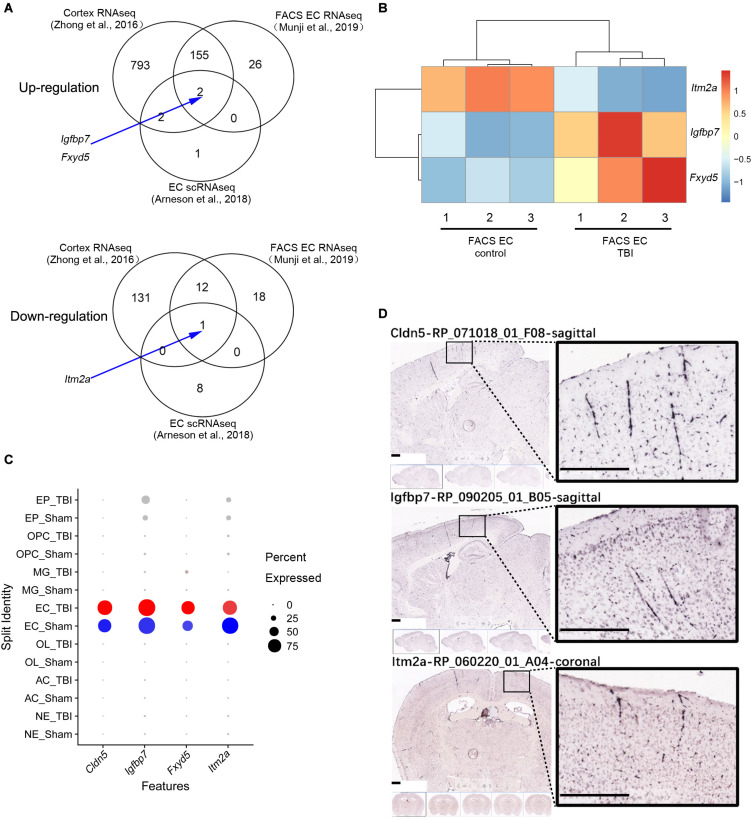
Analysis of the three shared DEGs. **(A)** Venn diagram illustrating the overlaps of the DEGs identified in the three studies, and the arrow indicate the three shared DEGs. **(B)** The details of the gene expression regulation of *Itm2a*, *Igfbp7*, and *Fxyd5* in the TBI study from the FACS sorted EC samples. **(C)** The dot plot illustrating the expression level and the expression percentage of *Cldn5*, *Igfbp7*, *Fxyd5*, and *Itm2a* in the major cell types identified from the scRNAseq study. *Cldn5* is a commonly used EC maker and used as a reference. The size of the dot represents the expression percentage in the cell type and the color intensity represents the expression level. The well-known EC marker *Cldn5* is used as a reference. EP, Ependymocyte; OPC, Oligodendrocyte progenitor cell; MG, Microglia; EC, endothelial cell; OL, Oligodendrocyte; AC, Astrocyte; NE, Neuron. **(D)** The vascular expression of *Igfbp7* and *Itm2a* genes expression using the Allen Brain Atlas database. Scale bar, 400 μm.

A comparison among the three different studies to identify differentially expressed genes (DEGs) in whole brain or brain EC is illustrated in Figure 2A. The DEGs of the three different studies are classified according to the up-regulated and down-regulated genes (Figure 2A). Totally, 1,135 different genes were identified by at least one study, and only three genes (*Igfbp7*, *Fxyd5*, and *Itm2a*) were consistently regulated in samples from TBI compared to control in all three studies. Among them, *Igfbp7* and *Fxyd5* are up-regulated, and *Itm2a* is down-regulated. Their detailed expression profile in the FACS-sorted EC samples is visualized in Figure 2B. All three genes showed EC specific expression in the single cell RNAseq study (Figure 2C). Expression of *Igfbp7* and *Itm2a* in the vasculature can further be confirmed in the Allen Brain Atlas database (Figure 2D).

To uncover whether alteration of the three genes is TBI specific or reflect a broader response to brain injury, we analyzed the expression of *Igfbp7/IGFBP7*, *Fxyd5/FXYD5*, and *Itm2a/ITM2A* in EC in response to stoke, seizure, and EAE in Munji’s study (Munji et al., 2019), as well as in the human glioblastoma vasculature in our previous study (Dieterich et al., 2012). Upregulation of *Fxyd5* in endothelial cells was observed in TBI and seizure. Down-regulation of *Itm2a* in endothelial cells was detected in TBI, seizure, and EAE (Supplementary Table 9). *Igfbp7/IGFBP7* expression was up-regulated in all these disease models as well as glioblastoma vasculature (Supplementary Table 9), suggesting that its up-regulation is a universal response in EC to pathological alterations rather than TBI-specific.

### IGFBP7 Is Upregulated in the Vasculature in Response TBI

**FIGURE 3:**
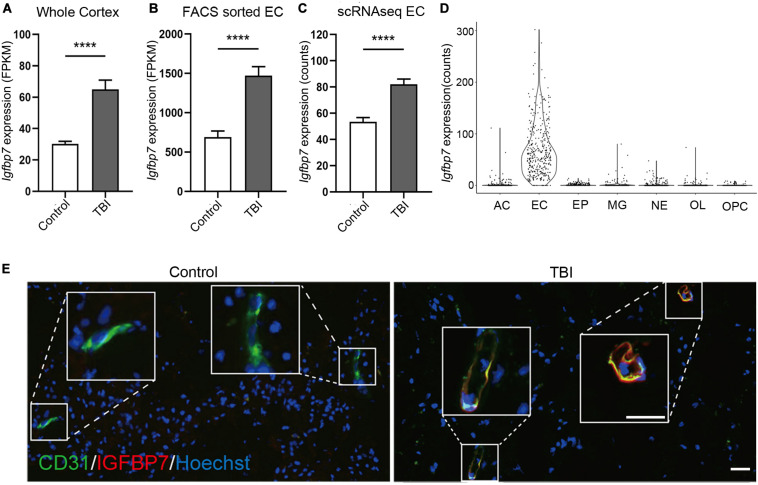
*Igfbp7/IGFBP7* is upregulated in the vasculature in response TBI. **(A–C)** The expression level of *Igfbp7* in the TBI and control groups from the three data sets. From left to right, whole cortex, FACS sorted EC RNAseq, and scRNAseq data sets. **** indicates *p* < 0.0001. **(D)** The expression level of *Igfbp7* in the main cell types from the scRNAseq study. AC, Astrocyte; EC, endothelial cell; EP, Ependymocyte; MG, Microglia; NE, Neuron; OL, Oligodendrocyte; OPC, Oligodendrocyte progenitor cell. **(E)** Immunofluorescence co-staining of CD31 (green) and IGFBP7 (red) in human samples. The TBI and control group were both operated from the right frontal. Scale bar, 50 μm.

Among the three commonly regulated genes in all three studies, *Igfbp7* were up-regulated in all datasets (Figures 3A–C), and also showed endothelial enriched expression from single cell data (Figure 3D). IGFBP7 has been suggested as a critical regulator for angiogenesis, vessel integrity, and endothelial adhesion molecule (Hooper et al., 2009; Komiya et al., 2014; Rai et al., 2015), which all associate with the pathogenesis of TBI. Thus, we focused on IGFBP7, a matrix bound secreted protein, which belongs to insulin-like growth factor binding protein (IGFBP) family. Immunofluorescence co-staining of CD31 and IGFBP7 in human samples clearly confirms up-regulation of IGFBP7 in vasculature in response to TBI (Figure 3E).

### TGFβ Induces *Igfbp7/IGFBP7* Expression in EC

**FIGURE 4:**
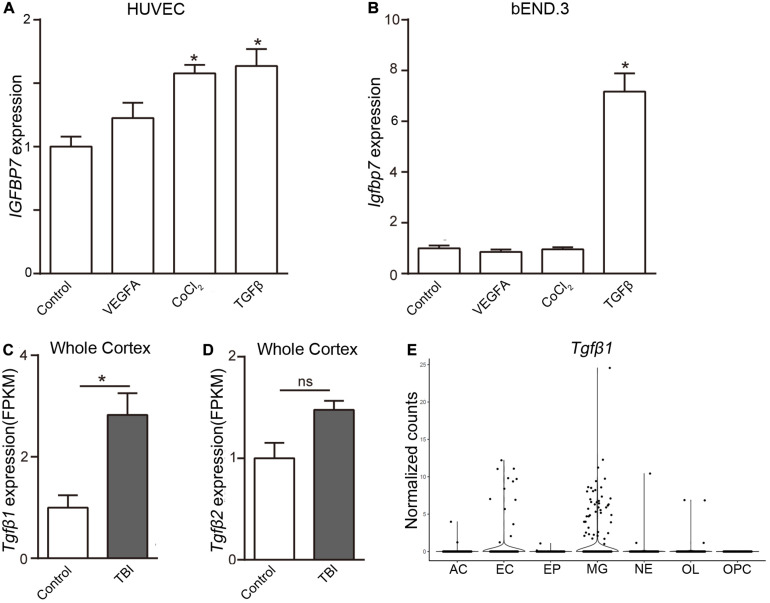
TGFβ induces the expression of *Igfbp7/IGFBP7* in endothelial cells. The normalized *Igfbp7/IGFBP7* expression levels in Control, VEGFA, CoCl_2_, and TGFβ stimulated HUVEC **(A)** and bEND.3 **(B)** cells are listed in the histogram, respectively. The expression levels of *TGF*β*1* **(C)** and *TGF*β*2* **(D)** from the whole cortex RNAseq data set. **(E)** The *TGF*β*1* expression in the main cell types from the scRNAseq study. AC, Astrocyte; EC, endothelial cell; EP, Ependymocyte; MG, Microglia; NE, Neuron; OL, Oligodendrocyte; OPC, Oligodendrocyte progenitor cell. * indicates *p* < 0.05.

To uncover the signal pathway mediating *IGFBP7* upregulation, we analyzed the expression of *Igfbp7/IGFBP7* in EC (bEND.3 cells and HUVECs) upon the stimulation of VEGFA and TGFβ, which can increase *IGFBP7* expression in breast cancer and glioblastoma vasculature respectively (Pen et al., 2008; Komiya et al., 2014). In addition, the effect of hypoxia, the most pronounced characteristic of brain injury causing pathogenesis, on *Igfbp7/IGFBP7* expression in EC was determined by stimulating cells with CoCl_2_, which induces hypoxia stimulation through HIF1α stabilization (Xu et al., 2015). VEGFA stimulation failed to upregulate *Igfbp7/IGFBP7* expression in either bEND.3 or HUVEC. Exposure of HUVECs to CoCl_2_ lead to a subtle upregulation of *IGFBP7* expression, while this effect could not be observed in bEND.3 cells (Figures 4A,B). Notably, *Igfbp7/IGFBP7* expression was significantly up-regulated in bEND.3 cells (7.2-folds) and HUVECs upon (1.6-folds) upon TGFβ stimulation (Figures 4A,B). In line with these findings, we observed a higher level of *TGF*β*1* in cortex from TBI compared to control in whole cortex RNAseq study (Zhong et al., 2016; Figure 4C), suggesting TGFβ signaling may potentiate vascular *IGFBP7* upregulation in TBI. Interestingly, there is no significant difference in *TGF*β*2* level in cortex between TBI and control (Figure 4D). To identify the main source of *TGF*β*1*, we have analyzed the *TGF*β*1* expression in single cell RNAseq dataset. *TGF*β*1* is mainly expressed in microglia and EC (Figure 4E). Based on these experiments *in vitro*, we proposed a potential mechanism that IGFBP7 up-regulation in endothelial cells in response to brain injury may be through TGFβ signaling. Experiments *in vivo* need to be further performed in the future to confirm the mechanism.

## Discussion

Brain vasculature plays critical roles in brain physiology and pathology. In our previous studies, we have illustrated transcriptome profiles in the normal vasculature (He et al., 2016, 2018; Vanlandewijck et al., 2018). In this study, we focus on the gene expression changes in the brain vascular EC under TBI condition. To cover common EC alteration in TBI pathogenesis, we integrated the results from two bulk RANseq studies (Zhong et al., 2016; Munji et al., 2019) and one single cell RNAseq study (Arneson et al., 2018). The controlled cortical impact (CCI) model was employed in Zhang’s and Munji’s studies to generate focal injury, while the fluid percussion injury (FPI) model was used in Arneson’s study to induce mixed injury. All studies applied the mouse model and brain RNA expression analyses were performed at 24 h after injury. In the comparison of regulated genes from the three studies, despite the significant difference due to sample compositions and techniques, we identified three genes that were consistently regulated in all three analyses (*Igfbp7*, *Fxyd5*, and *Itm2a*).

IGFBP7 is highly expressed in the vasculature during development in the central nervous system. But, in adults, the IGFBP7 expression was reported to be only restricted to smooth muscle cells covering large vessels and choroid plexus vasculature, which is characterized by limited BBB property and high permeability (Hooper et al., 2009; Bar et al., 2020). Increased IGFBP7 expression in vasculature was observed in brain pathological conditions including glioblastoma and stroke, as well as other types of tumors (Hooper et al., 2009; Dieterich et al., 2012; Buga et al., 2014; Komiya et al., 2014). In this study, we showed that *Igfbp7/IGFBP7* is up-regulated in the vasculature in both TBI mice models and surgical samples from patients with brain injury. Our results together with previous findings support the notion that *Igfbp7/IGFBP7* may be a general marker of vasculature in response to pathological conditions in the brain. A higher level of TGFβ and hypoxia was observed in the brain tissue after TBI (Park et al., 2009), and they can sufficiently upregulate *Igfbp7/IGFBP7* expression in EC, which may explain the molecular mechanism for *Igfbp7/IGFBP7* up-regulation in vasculature in response to TBI.

The role of IGFBP7 on pathogenesis of brain injury remains largely unknown. Exposure of EC to IGFBP7 leads to stress fiber formation and disorganization of VE-cadherin mediated junctions, resulting in increased vascular permeability (Komiya et al., 2014), which indicates a role of IGFBP7 on BBB breakdown. In addition, stimulation of brain EC with IGFBP7 upregulated E-selectin, a crucial molecule in immune cell recruitment (Rai et al., 2015), indicating that IGFBP7 may regulate neuroinflammation in response to brain injury.

Emerging studies convincingly showed that the injury vasculature attempts to undergo repair by inducing angiogenesis (Park et al., 2009). IGFBP7 can act as both pro- and anti-angiogenic factors (Hooper et al., 2009; Pen et al., 2011; Tamura et al., 2009, 2014; Komiya et al., 2014). IGFBP7 could block VEGFA-induced tube formation, EC migration, proliferation, and vascular permeability (Tamura et al., 2009, 2014). In contrast, other studies suggested that IGFBP7 promotes angiogenesis by increasing EC adhesion and VEGFA bioavailability (Hooper et al., 2009; Komiya et al., 2014). Thus, the contradictory effect of IGFBP7 on angiogenesis in different systems may depend on cues in the microenvironment, such as distinct regional composition of ECM (Hooper et al., 2009). Whether IGFBP7 contributes to vascular repair by regulating angiogenesis after brain injury deserves further investigation.

Taken together, our study reveals the key molecular alteration of EC and identifies IGFBP7 as a potential biomarker of vasculature in response to brain injury.

## Data Availability Statement

The datasets generated for this study can be found in the online repositories. The names of the repository/repositories and accession number(s) can be found in the article/Supplementary Material.

## Ethics Statement

The studies involving human participants were reviewed and approved by the ethics committee, Tianjin Medical University General Hospital. The patients/participants provided their written informed consent to participate in this study.

## Author Contributions

LH and LZ conceived the project. JW, YX, LZ, and ZZ performed the experiments. JW, XD, JT, FY, QH, QC, LZ, and LH analyzed the data. LZ and LH wrote the manuscript with significant input from JW and YX. All authors reviewed and approved the final manuscript.

## Conflict of Interest

The authors declare that the research was conducted in the absence of any commercial or financial relationships that could be construed as a potential conflict of interest.

## Footnotes

1 www.ncbi.nlm.nih.gov/sra

2 http://mccarrolllab.org/dropseq/

3 www.gsea-msigdb.org

## Supplementary Material

Supplementary Figure 1 Clustering and annotation of the 6,351 cells from the single cell RNAseq study. **(A)** A Uniform Manifold Approximation and Projection (UMAP) overview of the whole dataset with main cell type annotated. OPC, Oligodendrocyte progenitor cell; EC, endothelial cell. **(B)** The detailed expression levels of typical maker genes to illustrate the main cell types. The size of the dot represents the expression percentage in the cell type and the color represents the expression level. Cluster 5 displays unique expression of EC markers (*Pecam1*, *Cldn5*, and *Flt1*) and is selected as EC cluster for downstream analysis. Cluster 8 shows lower expression of EC markers, but with specific expression of mural cells markers (*Pdgfrb*, *Acta2*, and *Kcnj8*), suggesting that it is likely an EC-contaminated mural cell cluster.
