# Supplementary figures and images for: An Integrated Transcriptome Analysis Reveals IGFBP7 Upregulation in Vasculature in Traumatic Brain Injury
Source: Front Genet. 2021 Jan 11;11:599834. doi: 10.3389/fgene.2020.599834 (PMC7831608; doi:10.3389/fgene.2020.599834)

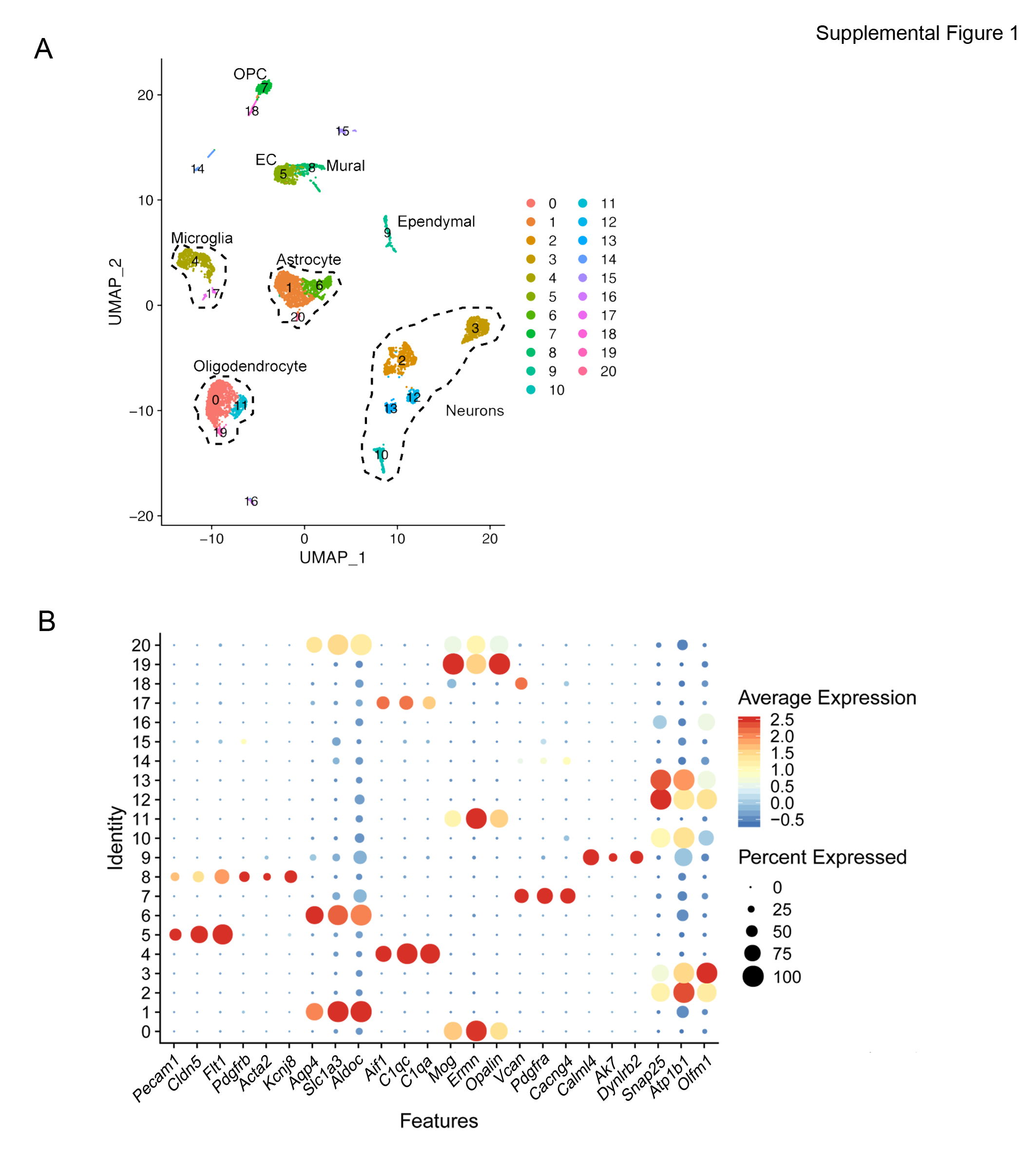

Supplement: Supplementary Figure 1 — Clustering and annotation of the 6,351 cells from the single cell RNAseq study. (A) A Uniform Manifold Approximation and Projection (UMAP) overview of the whole dataset with main cell type annotated. OPC, Oligodendrocyte progenitor cell; EC, endothelial cell. (B) The detailed expression levels of typical maker genes to illustrate the main cell types. The size of the dot represents the expression percentage in the cell type and the color represents the expression level. Cluster 5 displays unique expression of EC markers (Pecam1, Cldn5, and Flt1) and is selected as EC cluster for downstream analysis. Cluster 8 shows lower expression of EC markers, but with specific expression of mural cells markers (Pdgfrb, Acta2, and Kcnj8), suggesting that it is likely an EC-contaminated mural cell cluster. [file Image_1.JPEG]
